# Comparison of Chemical Components, Anti‐Inflammatory Effect and Cytotoxicity to Cancer Cells of Essential Oils From Different Parts of *Celosia argentea*

**DOI:** 10.1002/cbdv.71885

**Published:** 2026-10-30

**Authors:** Gia‐Buu Tran, Ty Viet Pham, Thao Xuan Hoang, Nguyen Hoai Nguyen, Bich Hang Do

**Affiliations:** ^1^ Research Group in Pharmaceutical and Biomedical Sciences, Faculty of Pharmacy Ton Duc Thang University Ho Chi Minh City Vietnam; ^2^ Faculty of Chemistry University of Education, Hue University Hue City Vietnam; ^3^ Faculty of Biology University of Education Hue University Hue City Vietnam; ^4^ Faculty of Biotechnology Ho Chi Minh City Open University Ho Chi Minh City Vietnam

**Keywords:** anti‐inflammatory effect, *Celosia argentea*, cytotoxicity to cancer cells, chemical components, essential oil

## Abstract

*Celosia argentea*, a therapeutic plant belonging to the *Celosia* genus, has been used in the treatment of various diseases. This study isolated essential oil (EO) from stems (CASO), leaves (CALO) and flowers (CAFO) which demonstrated that palmitic acid (16.93%), *cis*‐phytol (14.97%), hexahydrofarnesyl acetone (13.71%) were the main components in CAFO, *α*‐cadinene (44.42%), *cis*‐phytol (20.73%), linalool (8.86%) in CALO and (3*Z*)‐hexen‐1‐ol (41.90%), *n*‐hexanol (38.52%), (2*E*)‐hexenal (7.31%) in CASO. The main compounds in *C. argentea* EOs bound to inflammatory proteins, including inducible nitric oxide synthase and cyclooxygenase‐2, with scores ranging from −4.6 kcal/mol to −9.0 kcal/mol. Especially, *α*‐cadinene exhibited the best binding affinity toward iNOS (−9.0 kcal/mol), while demonstrating a binding affinity for COX‐2 (−8.3 kcal/mol) comparable to that of the positive controls. In an in vitro assay, CASO and CALO effectively inhibited nitric oxide production, exhibiting 92.53% ± 2.34% and 92.44% ± 1.95% inhibition at 100 µg/mL and IC_50_ values of 9.18 ± 0.65 µg/mL and 15.94 ± 1.18 µg/mL, respectively. Additionally, CASO and CALO exhibited cytotoxicity against cancer cells, with IC_50_ values of 7.39 ± 0.29 to 32.53 ± 4.04 µg/mL. These findings provide preliminary evidence of the anti‐inflammatory and cytotoxic potential of *C. argentea* EOs and support the need for further investigation of its biological activities and underlying mechanisms.

## Graphical abstract

*Celosia argentea* essential oil was investigated for its chemical composition and biological activities. GC–MS analysis identified its major constituents, while molecular docking was performed to explore their interactions with inflammation‐related targets. The essential oil exhibited anti‐inflammatory activity through inhibition of nitric oxide production and demonstrated cytotoxic effects against cancer cells, supporting its potential for further pharmaceutical investigation.

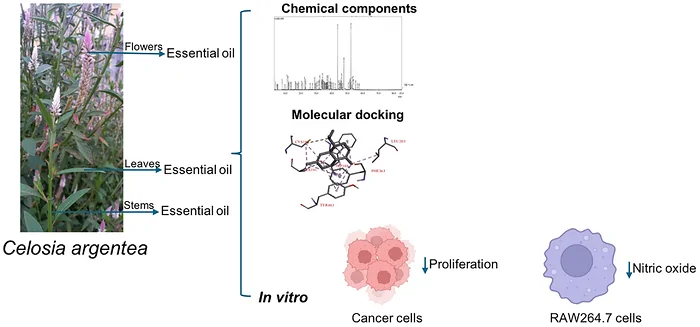

## 1 Introduction

*Celosia argentea* L., known as cockscomb, belongs to the *Celosia* genus and the Amaranthaceae family. This plant is widely distributed throughout subtropical and temperate areas. This plant, including the seed, leaf, and stem, was traditionally used to treat various diseases such as fever, diarrhea, inflammation, eye diseases, ulcers, to protect the liver, and to stop bleeding [1]. The seed, also called Celosiae semen, is a valuable medicine that appears in many traditional folk remedies. The bioactivity of *C. argentea* derived from interesting components, including saponins (celosins, cristatin), cyclopeptides (moroidin, celogentins, celogenamids), phenols (lutin, epigallocatechin, gallic acid, caffeic acid, rosmarinic acid, quercetin, 4‐O‐β‐D‐apifuranosyl‐(1→2)‐β‐D‐glucopyranosyl‐2‐hydroxy‐6‐methoxyacetophenone), fatty acids (arachic acid, arachidonic acid, linolenic acid, linoleic acid), amino acids and minerals, distributed mainly in the seed [2, 3, 4, 5, 6, 7]. The flower of *C. argentea* was a rich source of betalain, an effective antioxidant agent [8]. The hepatoprotective effect, antifungal and antibacterial activities against both Gram‐positive and Gram‐negative bacteria, wound‐healing, eye‐protective effect, anti‐inflammatory, anti‐cancer, anti‐diarrhea, anti‐diabetic, and antiurolithiatic activities were discovered in the *C. argentea* plant [9, 10]. The extracts from leaves, stems, and flowers of *C. argentea* exhibited antioxidant activity in vitro and in vivo models [8, 11, 12]. Saponins and glycosides isolated from *C. argentea* seed attenuate neural damage by increasing cell viability, decreasing reactive oxygen species generation, and inhibiting cell apoptosis in the tested cells [13].

Essential oils (EOs), isolated from aromatic plants and composed of volatile compounds, have attracted attention recently due to their diverse bioactivities, including antimicrobial, anti‐inflammatory, anti‐cancer, antioxidant, and neuroprotective properties [14]. The bioactivity of EOs might be derived from their main components such as terpenes (sesquiterpenes, diterpenes, monoterpenes), sometimes from the interaction between minority compounds [15]. Due to interesting bioactivity, EO has been widely used in industry, including cosmetics, food (preservatives, flavorings, packaging), pharmaceuticals (drug delivery, medicine), agriculture (pesticides, animal feed), and cleaning (solvents, degreasers) [16].

*C. argentea* is a medicinal plant that has been demonstrated to possess diverse bioactivities. However, most previous studies have focused on extracts, whereas research on the EO of this plant remains limited. Additionally, comparable investigations of EOs obtained from different parts of *C. argentea* would provide valuable insights into the chemical components and bioactivity of each plant part. Therefore, this is the first study to isolate EOs from different parts of the plant, including stems, leaves, and flowers. The chemical composition, anti‐inflammatory, and anticancer activities were evaluated in vitro and in silico.

## 2 Material and Methods

### 2.1 Plant Material Collection and Essential Oil Extraction

Plant samples of *C. argentea* were gathered on September 11, 2025, from coordinates 16°27'59''N, 107°33'29''E in Hue City, Vietnam. Identification was confirmed by Dr. Thao Xuan Hoang from Hue University. The voucher specimens, designated CA‐KL‐2025, are preserved in the herbarium at the Faculty of Chemistry, University of Education, Hue University, Vietnam. Collection was carried out in early autumn (September), during the local rainy season of central Vietnam, when the plant was at the flowering stage; flowers, mature leaves, and stems were all harvested from reproductive‐stage plants at this single time point, so that the three EOs compared in this study derive from the same phenological stage.

For EO isolation, fresh samples of flowers, leaves, and stems (250 g per part, with three replicates each) were washed, cut into segments of about 6–8 mm, and placed in a Clevenger‐type apparatus equipped with a 2000 mL round‐bottom flask. The flask contained 1500 mL of deionized water and was heated to 110°C via a heating mantle. The hydrodistillation process lasted 5 h under atmospheric pressure, with reflux, to obtain the EOs from each plant part. Following extraction, the oils were dehydrated using anhydrous sodium sulfate, filtered, and kept in vials at 5°C pending analysis. All procedures were replicated three times.

### 2.2 Volatile Compounds Analysis

The EOs from *C. argentea* flowers (CAFO), leaves (CALO), and stems (CASO) were examined for volatile constituents using a Shimadzu GC‐MS‐QP2010 Plus system (Kyoto, Japan), combining gas chromatography and mass spectrometry. Analysis adhered to established protocols from prior studies, ensuring reliable compound identification [17, 18, 19]. The instrument featured a fused silica Equity‐5 capillary column (30 m × 0.25 mm i.d., 0.25 µm film thickness; Supelco, USA). Operating conditions included helium carrier gas at 1.2 mL/min, injector and interface temperatures at 280°C, and a temperature ramp starting at 60°C (held 2 min), rising to 240°C at 3°C/min (held 10 min), then to 280°C at 5°C/min (held 30 min). Samples were injected at 1.0 µL with a 40:1 split ratio and 93.2 kPa inlet pressure. Mass spectra were acquired at 70 eV ionization voltage, 0.82 kV detector voltage, 40–500 amu scan range, and 0.5 scans/s. Retention indices (RI) were calculated by co‐injection with C_7–_C_40_ *n*‐alkanes using the generalised temperature‐programmed retention‐index equation of Van Den Dool and Kratz ^x^: RI = 100 × [n + (*t*_x_—*t*_n_)/(*t*_n _+ 1 ‐ *t*_n_)], where *t*_x_ is the retention time of the analyte and *t*_n_ and *t*_n _+ 1 are the retention times of the *n*‐alkanes eluting immediately before and after the analyte, respectively [20]. Compounds were identified by comparing RI values to literature data and matching fragmentation patterns to the NIST 20, and WILEY 7 libraries. The identifications were further cross‐checked against the mass‐spectral and RI compendium of Adams [21]. Relative abundances were determined from peak areas in the total ion chromatogram.

### 2.3 Nitric Oxide Inhibition Assay

The inhibitory effect of *C. argentea* EOs on nitric oxide (NO) production in activated macrophages was assessed according to a previously described method [22]. RAW 264.7 murine macrophage cells (RRID: CVCL_0493, supplied by Institute of Biology, Vietnam Academy of Science and Technology) were subcultured several times until the cells were stable. The healthy cells were seeded in 96‐well plates, incubated for 24 h, then treated with EOs at concentrations of 100, 20, 4, and 0.8 µg/mL. After 2 h of treatment, the cells were treated with lipopolysaccharide (LPS; Life Technologies, Inc., USA) and incubated further for an additional 24 h. NO released was measured using the Griess reagent (Promega Corporation, USA) according to the kit protocol. Absorbance was measured at 540 nm using a BioTek ELx800 microplate reader (Thermo–Fisher Scientific, MA, USA). Dexamethasone Sigma Chemical Co., USA) at concentrations of 39.25, 7.85, 1.57, and 0.31 µg/mL was used as a positive control.

The percentage inhibition of NO production was calculated using the following formula:

$$\%\;\text{Inhibition}=(1-OD_{\text{sample}}/OD_{\text{LPS}})\times 100\%$$

IC_50_ values were determined using TableCurve 2D version 4 software.

Cell viability of RAW 264.7 macrophages following *C. argentea* EOs treatment was evaluated using the MTT assay. The cells were treated with MTT, and the formazan crystals were solubilized by dimethyl sulfoxide. The absorbance was measured at 540 nm using a microplatespectrophotometer. Cell viability was calculated using the following formula:

$$\%\;\text{Viability}=\left(OD_{\text{sample}}/OD_{\text{control}}\right)\times 100$$

### 2.4 Molecular Docking

The binding affinity of major constituents of *C. argentea* EOs with proteins related to inflammation, including cyclooxygenase‐2 (COX‐2; PDB ID: 1CVU) and inducible nitric oxide synthase (iNOS; PDB ID: 3NQS), was identified using AutoDock Tools according to a previous study [23]. The ligand binding sites on target proteins were positioned at the center of the reference ligand, with a grid box size of x = 28, y = 28, z = 28. The docking procedure was validated by redocking the co‐ligand into the corresponding binding sites. Docking poses and protein–ligand interactions were visualized in both three‐dimensional and two‐dimensional representations using Discovery Studio Visualizer 2024 and PyMOL version 3.1.3, respectively.

### 2.5 Viability of Cells

Immortalized human T lymphocyte (Jurkat, RRID: CVCL_0065), adenocarcinomic human alveolar basal epithelial (A549, RRID: CVCL_0023) and human hepatocellular carcinoma (Huh‐7, RRID: CVCL_0336) (supplied by Institute of Biology, Vietnam Academy of Science and Technology) cells were maintained in Dulbecco's Modified Eagle's Medium (DMEM) supplemented with L‐glutamine, sodium pyruvate, NaHCO_3_, penicillin–streptomycin, and 10% fetal bovine serum (FBS) under a humidified atmosphere of 5% CO_2_ at 37°C. After reaching optimal growth, viable cells were seeded into 96‐well plates and allowed to stabilize for 18–20 h.

Cells were then treated with various concentrations of *C. argentea* EOs (100, 20, 4, and 0.8 µg/mL) and incubated for 48 h at 37°C. The viability of cells was assessed using the MTT assay, following the previous protocol [24]. Ellipticine (10, 2, 0.4, and 0.08 µg/mL) and 0.5% DMSO were used as positive and negative controls, respectively. The IC_50_ values were calculated using TableCurve 2D version 4 software.

### 2.6 Data Analysis

All data are expressed as the mean ± standard deviation of three independent experiments. Graphical presentation and statistical analyses were performed using GraphPad Prism software (version 9.0.0.121; GraphPad Software, Inc., San Diego, CA, USA). Statistical differences between groups were evaluated using one‐way ANOVA, and values of *p* < 0.05 were considered statistically significant.

## 3 Results and Discussion

### 3.1 Chemical Composition of EOs From Different Organs of *C. argentea*

The *C. argentea* EOs were analyzed, revealing 83 compounds that accounted for 96.75–99.86% of the total peak area (Table 1). GC chromatograms for these samples are provided (Figure 1), with peaks labeled for each identified compound to illustrate the separation and profiling process, thereby enhancing the visualization of compositional differences.

**TABLE 1:** Essential oil compositions from different parts of *C. argentea*.

No	RT	Compound	RI _E_	RI _L_	CAFO	CALO	CASO	Classification
1	3.81	*n*‐Hexanal	800	801	0.49 ± 0.00	N/A	0.24 ± 0.00	NT
2	4.89	(2*E*)‐Hexenal	847	846	0.19 ± 0.00	0.44 ± 0.00	**7.31 ± 0.02**	NT
3	4.96	(3*Z*)‐Hexen‐1‐ol	851	850	0.18 ± 0.00	3.27 ± 0.01	**41.90 ± 0.11**	NT
4	5.21	(2*Z*)‐Hexenol	861	859	N/A	0.34 ± 0.00	N/A	NT
5	5.25	*n*‐Hexanol	863	863	0.24 ± 0.00	0.65 ± 0.00	**38.52 ± 0.10**	NT
6	8.03	Benzaldehyde	958	952	1.15 ± 0.01	N/A	0.39 ± 0.00	NT
7	9.72	(3*E*)‐Hexenyl acetate	1006	1001	N/A	1.44 ± 0.00	N/A	NT
8	9.88	(2*E*,4*E*)‐Heptadienal	1010	1005	0.19 ± 0.00	N/A	N/A	NT
9	11.19	Benzene acetaldehyde	1042	1036	1.88 ± 0.00	N/A	0.58 ± 0.00	NT
10	11.80	*γ*‐Terpinene	1057	1054	0.24 ± 0.00	N/A	N/A	MH
11	12.15	Acetophenone	1065	1059	0.18 ± 0.00	N/A	N/A	NT
12	12.24	(2*E*)‐Octen‐1‐ol	1067	1060	0.38 ± 0.00	N/A	N/A	NT
13	12.35	*n*‐Octanol	1070	1063	0.55 ± 0.00	N/A	N/A	NT
14	13.59	Linalool	1100	1100	0.33 ± 0.00	**8.86 ± 0.02**	N/A	OM
15	13.78	*n*‐Nonanal	1104	1100	0.43 ± 0.00	N/A	N/A	NT
16	15.60	Camphor	1145	1141	N/A	N/A	0.51 ± 0.00	OM
17	16.59	Borneol	1167	1165	0.74 ± 0.00	N/A	N/A	OM
18	16.79	*cis*‐Linalool oxide (pyranoid)	1172	1170	0.38 ± 0.00	N/A	N/A	OM
19	17.67	*α*‐Terpineol	1191	1186	0.34 ± 0.00	4.37 ± 0.01	N/A	OM
20	18.31	*n*‐Decanal	1206	1201	0.20 ± 0.00	N/A	N/A	NT
21	18.63	*trans*‐Pulegol	1213	1213	0.62 ± 0.00	0.65 ± 0.00	N/A	OM
22	19.34	Nerol	1229	1227	N/A	1.79 ± 0.01	N/A	OM
23	20.52	Geraniol	1255	1249	0.67 ± 0.01	**6.10 ± 0.01**	N/A	OM
24	21.23	Geranial	1271	1264	0.49 ± 0.00	N/A	N/A	OM
25	22.50	*n*‐Tridecane	1300	1300	1.47 ± 0.01	N/A	N/A	NT
26	23.12	*ρ*‐vinyl‐Guaiacol	1314	1309	0.59 ± 0.00	0.98 ± 0.00	N/A	OM
27	23.24	4‐hydroxy‐Cryptone	1317	1314	0.83 ± 0.01	N/A	N/A	OM
28	23.60	(2*E*,4*E*)‐Decadienol	1325	1319	N/A	1.04 ± 0.00	N/A	NT
29	26.16	Geranyl acetate	1384	1379	0.28 ± 0.00	N/A	N/A	OM
30	27.71	(*E*)‐Caryophyllene	1421	1417	0.31 ± 0.00	N/A	N/A	SH
31	29.05	*α*‐Humulene	1454	1452	1.81 ± 0.00	N/A	N/A	SH
32	29.41	dehydro‐Aromadendrane	1463	1460	0.36 ± 0.00	N/A	N/A	SH
33	29.91	*trans*‐Cadina‐1(6),4‐diene	1475	1475	0.39 ± 0.00	N/A	N/A	SH
34	30.45	(*E*)‐*β*‐Ionone	1488	1487	0.82 ± 0.00	N/A	1.43 ± 0.01	OS
35	31.38	Premnaspirodiene	1511	1505	0.74 ± 0.00	N/A	N/A	SH
36	31.98	*δ*‐Cadinene	1526	1522	0.60 ± 0.00	N/A	N/A	SH
37	32.36	*α*‐Cadinene	1536	1537	2.08 ± 0.01	**44.42 ± 0.09**	N/A	SH
38	33.52	Germacrene B	1565	1559	0.80 ± 0.00	N/A	N/A	SH
39	33.68	1,5‐epoxysalvial‐4(14)‐ene	1570	1561	1.36 ± 0.00	N/A	N/A	OS
40	33.79	Dendrolasin	1572	1570	0.39 ± 0.00	N/A	N/A	OS
41	34.13	Spathulenol	1581	1577	2.44 ± 0.01	N/A	1.10 ± 0.01	OS
42	34.36	Caryophyllene oxide	1587	1582	1.24 ± 0.00	N/A	N/A	OS
43	34.84	Guaiol	1599	1600	0.78 ± 0.00	N/A	N/A	OS
44	35.16	Curzerenone	1608	1605	N/A	N/A	0.61 ± 0.00	OS
45	35.27	(2*E*)‐Dodecenyl acetate	1611	1608	0.32 ± 0.00	N/A	N/A	OS
46	35.36	*cis*‐Isolongifolanone	1613	1612	0.63 ± 0.00	N/A	N/A	OS
47	35.56	Isolongifolan‐7‐*α*‐ol	1618	1618	0.27 ± 0.01	N/A	N/A	OS
48	36.06	Eremoligenol	1632	1629	0.35 ± 0.00	N/A	N/A	OS
49	36.42	*cis*‐Cadin‐4‐en‐7‐ol	1641	1635	0.30 ± 0.00	N/A	N/A	OS
50	36.57	epi‐*α*‐Muurolol	1645	1640	0.71 ± 0.00	N/A	N/A	OS
51	36.73	*α*‐Muurolol (= Torreyol)	1650	1644	0.22 ± 0.00	N/A	N/A	OS
52	37.05	Valerianol	1658	1656	1.95 ± 0.01	N/A	N/A	OS
53	37.20	Selin‐11‐en‐4‐*α*‐ol	1662	1658	0.53 ± 0.00	N/A	N/A	OS
54	37.37	Allohimachalol	1667	1661	0.24 ± 0.00	N/A	N/A	OS
55	37.66	*n*‐Tetradecanol	1675	1671	0.87 ± 0.00	N/A	N/A	NT
56	37.78	Mustakone	1678	1676	0.75 ± 0.00	N/A	N/A	OS
57	38.29	Acorenone	1692	1692	N/A	N/A	0.61 ± 0.00	OS
58	38.60	Caryophyllene acetate	1700	1701	0.63 ± 0.00	N/A	N/A	OS
59	39.15	*n*‐Pentadecanal	1715	1715	1.39 ± 0.00	N/A	N/A	NT
60	39.46	(2*Z*,6*E*)‐Farnesol	1724	1722	0.27 ± 0.01	N/A	N/A	OS
61	39.69	*iso*‐Longifolol	1730	1728	0.65 ± 0.00	N/A	N/A	OS
62	40.89	Benzyl benzoate	1764	1759	0.62 ± 0.00	N/A	N/A	NT
63	42.16	*n*‐Octadecane	1800	1800	0.27 ± 0.00	N/A	N/A	NT
64	43.77	Hexahydrofarnesyl acetone	1848	1845	**13.71 ± 0.01**	N/A	1.41 ± 0.00	OS
65	44.31	*cis*‐Thujopsenic acid	1864	1863	0.41 ± 0.00	N/A	N/A	OS
66	44.47	Benzyl salicylate	1868	1864	0.94 ± 0.00	N/A	N/A	NT
67	45.22	(5*E*,9*Z*)‐Farnesyl acetone	1891	1886	0.33 ± 0.00	N/A	N/A	OS
68	45.55	*n*‐Nonadecane	1900	1900	1.07 ± 0.00	N/A	N/A	NT
69	46.20	Totarene	1921	1922	1.11 ± 0.00	N/A	N/A	DH
70	46.44	Methyl palmitate	1928	1928	2.31 ± 0.01	N/A	0.93 ± 0.00	NT
71	47.13	Phytol	1949	1942	0.33 ± 0.00	N/A	0.46 ± 0.00	OD
72	47.94	Palmitic acid	1974	1959	**16.93 ± 0.02**	N/A	1.11 ± 0.01	NT
73	50.75	Ocenol	2064	2060	0.34 ± 0.00	N/A	N/A	NT
74	51.74	Methyl linoleate	2096	2095	0.81 ± 0.00	N/A	N/A	NT
75	51.89	*n*‐Heneicosane	2101	2100	1.82 ± 0.01	N/A	N/A	NT
76	52.16	Methyl linolenate	2110	2103	0.24 ± 0.00	N/A	N/A	NT
77	52.35	*cis*‐Phytol	2117	2113	**14.97 ± 0.01**	**20.73 ± 0.04**	2.04 ± 0.01	OD
78	52.97	Oleic Acid	2138	2140	1.63 ± 0.01	N/A	N/A	NT
79	53.78	Ethyl linoleate	2165	2159	0.36 ± 0.00	N/A	N/A	NT
80	53.95	*cis*,*cis*,*cis*‐7,10,13‐Hexadecatrienal	2171	MS	N/A	4.78 ± 0.01	N/A	NT
81	55.45	Isophytol, acetate	2222	MS	0.85 ± 0.00	N/A	N/A	OD
82	56.65	7‐Hexyleicosane	2265	MS	0.23 ± 0.00	N/A	N/A	NT
83	57.68	*n*‐Tricosane	2301	2300	0.23 ± 0.00	N/A	N/A	NT
Total					96.75 ± 0.10	99.86 ± 0.18	99.17 ± 0.26	
Monoterpene hydrocarbon (MH)					0.24 ± 0.00	0.00 ± 0.00	0.00 ± 0.00	
Oxygenated monoterpene (OM)					5.27 ± 0.02	22.75 ± 0.05	0.51 ± 0.00	
Sesquiterpene hydrocarbon (SH)					7.09 ± 0.01	44.42 ± 0.09	0.00 ± 0.00	
Oxygenated sesquiterpene (OS)					29.30 ± 0.05	0.00 ± 0.00	5.16 ± 0.02	
Diterpene hydrocarbon (DH)					1.11 ± 0.00	0.00 ± 0.00	0.00 ± 0.00	
Oxygenated diterpene (OD)					16.15 ± 0.01	20.73 ± 0.04	2.50 ± 0.01	
Nonterpenic compounds (NT)					37.59 ± 0.07	11.96 ± 0.02	90.98 ± 0.24	

*Note*: RT, retention time; RI_E_, experimental retention indices; RI_L_, literature retention indices; MS, mass spectra obtained in electron ionization (EI) mode; N/A, not available. Bold: major compounds ( ≥ 6%). Compounds were identified based on agreement between experimental and literature RI together with mass spectral matching (NIST 20, WILEY 7 and Adams libraries) and, where available, comparison with reference EOs; for three compounds (entries 80–82) for which no literature RI value could be found, identification relied on mass spectral matching and comparison with reference EOs only, and MS is indicated in the RI_L_ column for these entries; identifications based on RI+MS agreement are therefore considered more reliable than the three MS‐only assignments.

**FIGURE 1:**
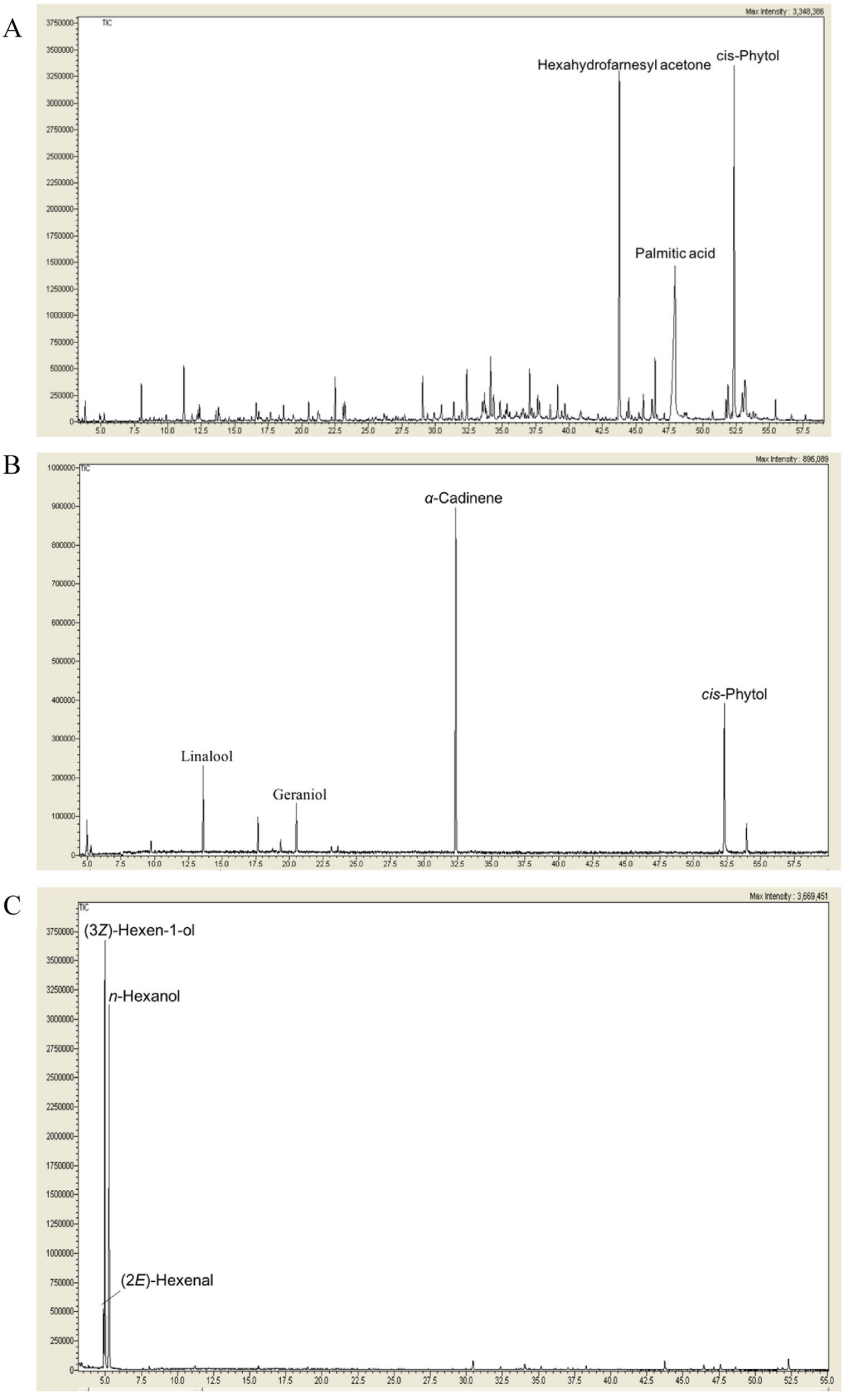
GC chromatogram of the essential oil from *C. argentea* flowers (A), leaves (B), and stems (C).

CAFO was yellow with a 0.02% (w/w, fresh weight basis) yield, containing 75 compounds that accounted for 96.75% of the total composition (Table 1). Non‐terpenic compounds were the dominant class (37.59%), followed by oxygenated sesquiterpenes (29.30%), oxygenated diterpenes (16.15%), sesquiterpene hydrocarbons (7.09%), oxygenated monoterpenes (5.27%), diterpene hydrocarbons (1.11%), and monoterpene hydrocarbons (0.24%). Prominent constituents included palmitic acid (16.93%), *cis*‐phytol (14.97%), hexahydrofarnesyl acetone (13.71%), spathulenol (2.44%), methyl palmitate (2.31%), and *α*‐cadinene (2.08%).

The CALO was yellow in color, with a yield of 0.04% (w/w, fresh weight basis). A total of 15 compounds were identified, accounting for 99.86% of the oil (Table 1). Sesquiterpene hydrocarbons were the dominant compounds (44.42%), followed by oxygenated monoterpenes (22.75%), oxygenated diterpenes (20.73%), and non‐terpenic compounds (11.96%). Major components were *α*‐cadinene (44.42%), *cis*‐phytol (20.73%), linalool (8.86%), geraniol (6.10%), *cis*,*cis*,*cis*‐7,10,13‐hexadecatrienal (4.78%), *α*‐terpineol (4.37%), and (3*Z*)‐hexen‐1‐ol (3.27%).

CASO yielded 0.06% (w/w, fresh weight basis) of yellow oil, with 16 compounds representing 99.17% of the total composition (Table 1). Nonterpenic compounds predominated (90.98%), accompanied by oxygenated sesquiterpenes (5.16%), oxygenated diterpenes (2.50%), and oxygenated monoterpenes (0.51%). Key compounds included (3*Z*)‐hexen‐1‐ol (41.90%), *n*‐hexanol (38.52%), (2*E*)‐hexenal (7.31%), and *cis*‐phytol (2.04%). Chemical structures of the major volatile compounds in CAFO, CALO, and CASO are expressed in Figure 2.

**FIGURE 2:**
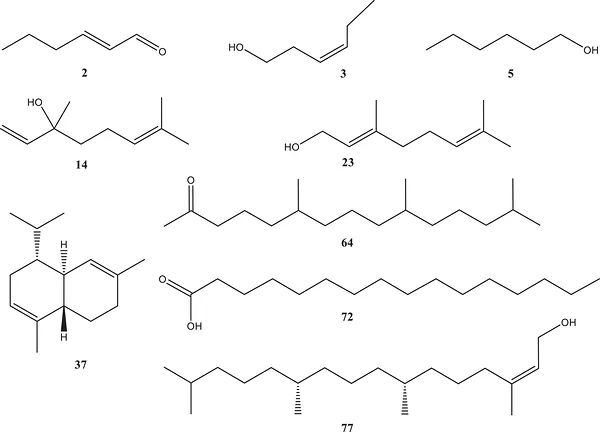
Chemical structures of the major volatile compounds (bold entries, Table 1) identified in *C. argentea* EOs.

A comparative study of *C. argentea* EOs reveals compositional differences, likely due to the metabolic functions of the different parts. Profiles of CAFO were more diverse (75 compounds) than CALO (15 compounds) and CASO (16 compounds), indicating greater complexity of biochemical processes in reproductive organs. There is an increase in the proportion of yield (CAFO, 0.02%; CALO, 0.04%; CASO, 0.06%), indicating that the volatile compounds are predominantly concentrated in the *C. argentea* stem. Additionally, significant variation in chemical class distributions was observed among plant parts. While CAFO contains comparable levels of terpenic and non‐terpenic compounds, CALO was enriched in terpenes, and CASO showed a high proportion of non‐terpenic compounds. Compounds such as (2*E*)‐hexenal, (3*Z*)‐hexen‐1‐ol, *n*‐hexanol, and *cis*‐phytol were detected in all three EOs, although their relative abundances differed, with high levels observed in CAFO. Such variations may be significant and need further ecological or pharmacological investigation.

Scientific literature on the chemical composition of EOs from *C. argentea* remains sparse. A study on Iranian‐sourced *C. argentea* aerial parts employed supercritical fluid extraction to identify 37 volatile compounds, with major constituents including cembrene (34.689%), palmitic acid (25.075%), 6,10,14‐trimethyl‐2‐pentadecanone (6.903%), *n*‐nonanal (4.520%), (*E*)‐caryophyllene (4.218%), pulegone (3.279%), *n*‐heneicosane (2.940%), *N*,*N*,4‐trimethylbenzenamine (2.894%), and geranyl acetone (2.077%) [25]. Soxhlet extraction of Ivorian *C. argentea* seeds with *n*‐hexane at 60°C revealed major linoleic acid (49.94%), oleic acid (22.34%), palmitic acid (19.13%), and stearic acid (7.02%) [26]. Related species, such as African *C. spicata* seeds extracted via Soxhlet with *n*‐hexane at 60–80°C, contained palmitic acid (29.84%) and linoleic acid (23.29%) as primary fatty acids, with arachidic acid at minimal levels (0.505%) [27]. Shared elements, such as palmitic acid and *cis*‐phytol across organs, align with Iranian aerial parts (palmitic acid at 25.075%) and Ivorian seeds (palmitic acid at 19.13%). Overall, the current investigation underscores organ‐specific volatile diversity, with shared motifs like *cis*‐phytol across parts and lower yields compared to prior extraction‐optimized studies, emphasizing the influence of geographical origin, plant organ, and methodological factors on EO composition.

### 3.2 Nitric Oxide Inhibition

To examine whether *C. argentea* EOs were able to reduce the NO release of macrophage cells, a Griess assay was performed. As presented in Table 2, cell viability exceeded 79% across all tested concentrations of EOs, indicating that these concentrations were appropriate for subsequent NO evaluation. Figure 3 demonstrated that three parts of *C. agentea* effectively inhibited NO production, with inhibition percentages ranging from 90.87% ± 2.49% to 92.53% ± 2.34% at 100 µg/mL. Among them, CASO was the most effective with an IC_50_ of 9.18 ± 0.65 µg/mL, which was approximately equal to that of the positive control, dexamethasone (4.75 ± 0.57 µg/mL). CALO exhibited higher activity than that of CAFO, with the IC_50_ values of 15.94 ± 1.18 and 33.06 ± 2.09 µg/mL, respectively.

**TABLE 2:** The viability of RAW264.7 cells treated with *C. argentea* EOs.

Conc. (µg/mL)	CAFO	CALO	CASO
100	87.61 + 1.08	79.95 + 1.57	79.93 + 1.49
20	89.4 + 1.12	87.67 + 2.07	89.43 + 0.83
4	NT	NT	NT
0.8	NT	NT	NT

*Note*: not tested. The cell viability of EOs was higher than 80% at 20 µg/mL; therefore, lower concentrations of EOs were not further evaluated.

**FIGURE 3:**
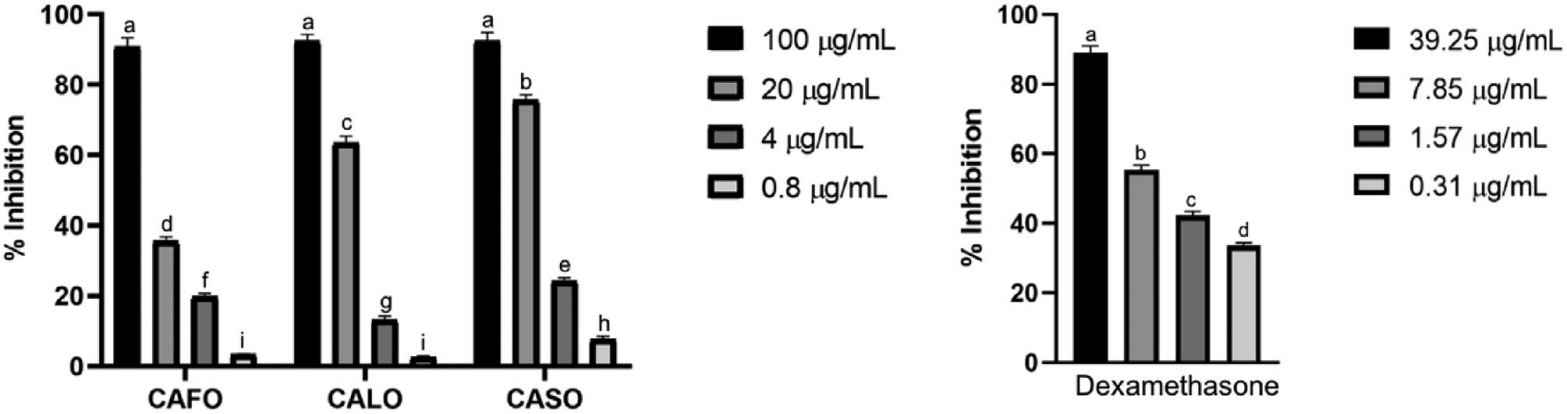
The inhibition of NO release in RAW264.7 cells. Dexamethasone was used as a positive control. One‐way ANOVA with Tukey's multiple comparison was used; different letters represent significant differences.

Compared with previous studies on the anti‐inflammatory effects of *C. argentea* extracts*, C. argentea* EOs appeared more effective. In particular, the acetone extract exhibited moderate activity, while the aqueous and methanol extracts were unable to reduce NO release from stimulated macrophage cells [28]. The flavonoid fraction from the alcoholic extract of the leaves of *C. argentea* at 10 mg/kg exhibited an anti‐inflammatory effect in vivo using carrageenan‐induced paw edema and cotton pellet‐induced granuloma models [29]. The leaf methanol extract of *C. argentea* reduced the edema of the mouse paw by 39.13% at 400 mg/kg *p.o* [30]. The major components of sesquiterpene hydrocarbons, oxygenated monoterpenes, oxygenated sesquiterpenes, and oxygenated diterpenes may explain the anti‐inflammatory activity of EOs [31, 32]. Sesquiterpenes reduced inflammation via downregulating the release of pro‐inflammatory mediators associated with PI3K/Akt and 5‐LOX signaling pathways [33]. Additionally, other EOs containing the same major compounds as those identified in *C. argentea* EOs have also been reported to exhibit anti‐inflammatory activity. EO rich in (3Z)‐hexen‐1‐ol, the main compound in CASO (41.9%), expressed anti‐inflammatory properties in a mouse model [34]. EO rich in δ‐cadinene, a sesquiterpene hydrocarbon found mostly in CALO (44.42%), reduced the edema in carrageenan‐induced models [35]. Several compounds with low percentages in the EOs but expressed high anti‐inflammation, including (*E*)‐caryophyllene, *α*‐humulene, geraniol, linalool, and spathulenol [36, 37, 38, 39, 40]. Furthermore, the synergistic interaction among plant‐derived bioactive compounds in EOs may enhance the activity of the EOs [41].

### 3.3 Molecular Docking

The binding of the main compounds in CAFO, CALO, and CASO with inflammatory proteins, including iNOS and COX‐2, was evaluated using AutoDock Vina. The docking protocol was validated by redocking the co‐ligand into the active site. The resulting RMSD values were below 2.0 Å, indicating good agreement between the experimental and predicted binding poses and confirming the reliability of the docking protocol (Table S1 and Figure S1). The docking results of the main compounds in *C. argentea* EOs with iNOS and COX‐2 indicated that all compounds bound to the tested proteins with docking scores ranging from ‐4.6 to ‐9.0 kcal/mol (Table 3 and Figure 4). Additionally, the compounds consisting of palmitic acid, cis‐phytol, hexahydrofarnesyl acetone, geraniol, and *n*‐hexanol formed hydrogen bonds with COX‐2, while no hydrogen bond was formed with iNOS. Among the compounds, *α*‐cadinene (the major constituent in CALO) exhibited the strongest binding affinity toward both iNOS (‐9.0 kcal/mol) and comparable affinity to COX‐2 (‐8.3 kcal/mol), relative to the positive controls. The in silico results complemented the in vitro findings, supporting the anti‐inflammatory activity of *C. argentea* EOs.

**TABLE 3:** The docking score of the main compounds of *C. argentea* EOs with iNOS and COX‐2.

Compound		Docking score	No. of H‐bonds	Residues
**iNOS (PDB: 3NQS)**				
Dexamethasone		−8.4	3	Arg 193, Ile 195, Gly 365
CAFO	Palmitic acid	−5.8	0	—
	*cis*‐Phytol	−6.1	0	—
	Hexahydrofarnesyl acetone	−6.2	0	—
CALO	***α*‐Cadinene**	**−9.0**	0	—
	Linalool	−6.3	0	—
	Geraniol	−6.4	0	—
CASO	(3*Z*)‐Hexen‐1‐ol	−5.0	0	—
	*n*‐Hexanol	−4.7	0	—
	(2*E*)‐Hexenal	−5.0	0	—
**COX‐2 (PDB: 1CVU)**				
	Diclofenac	−8.6	1	ARG 120
CAFO	Palmitic acid	−6.8	2	ASN 375, GLY 227
	Cis‐phytol	−7.4	1	GLY 526
	Hexahydrofarnesyl acetone	−6.9	1	SER 530
CALO	***α*‐Cadinene**	**−8.3**	0	—
	Linalool	−5.8	0	—
	Geraniol	−6.0	2	ASN 382, HIS 386
CASO	(3*Z*)‐Hexen‐1‐ol	−4.7	0	—
	*n*‐Hexanol	−4.6	2	ASN 375, GLY 533
	(2*E*)‐Hexenal	−5.0	0	—

FIGURE 4The docking picture of *C. argentea* EO compounds with iNOS and COX‐2.

**Figure:**
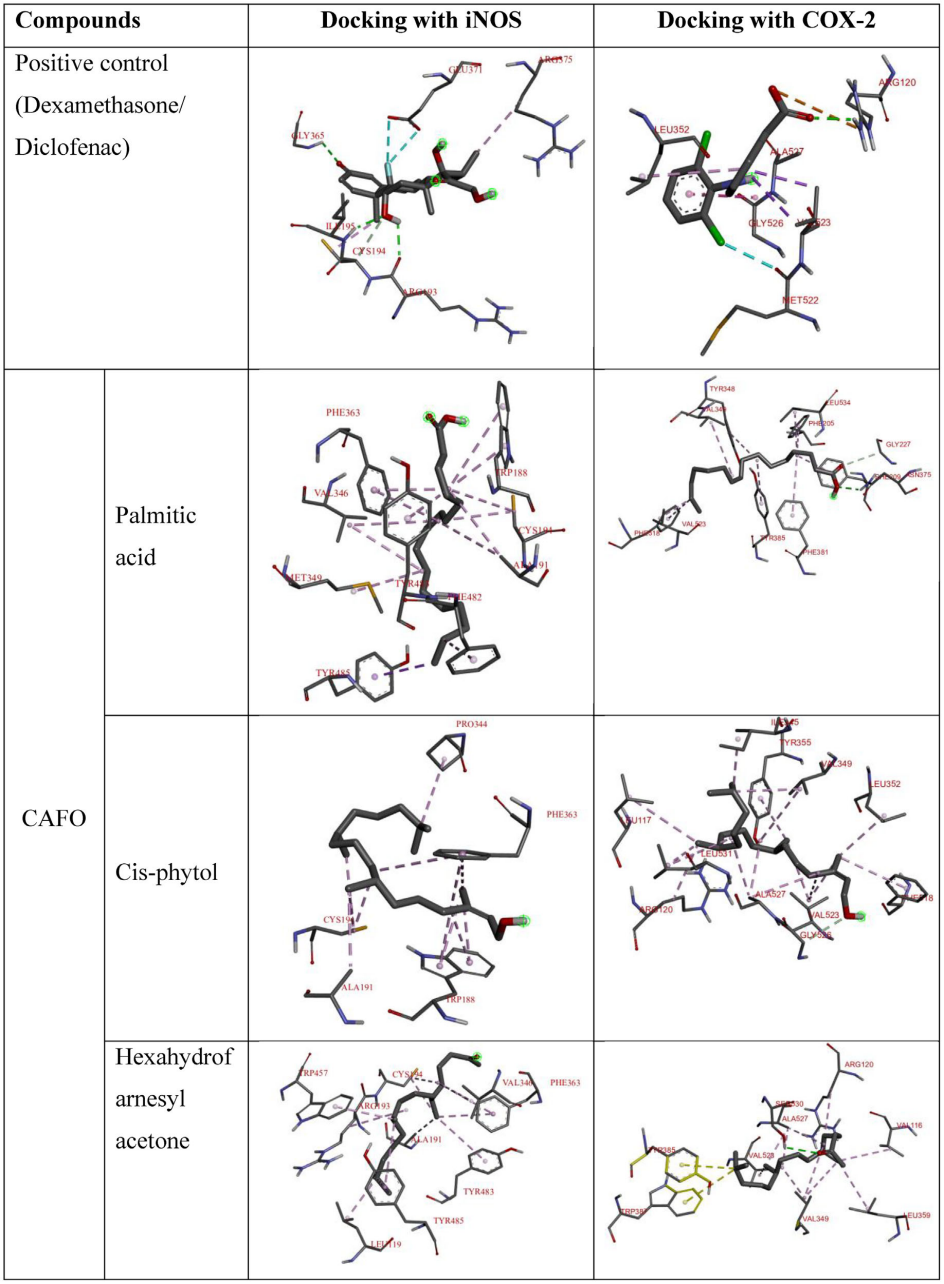

**Figure:**
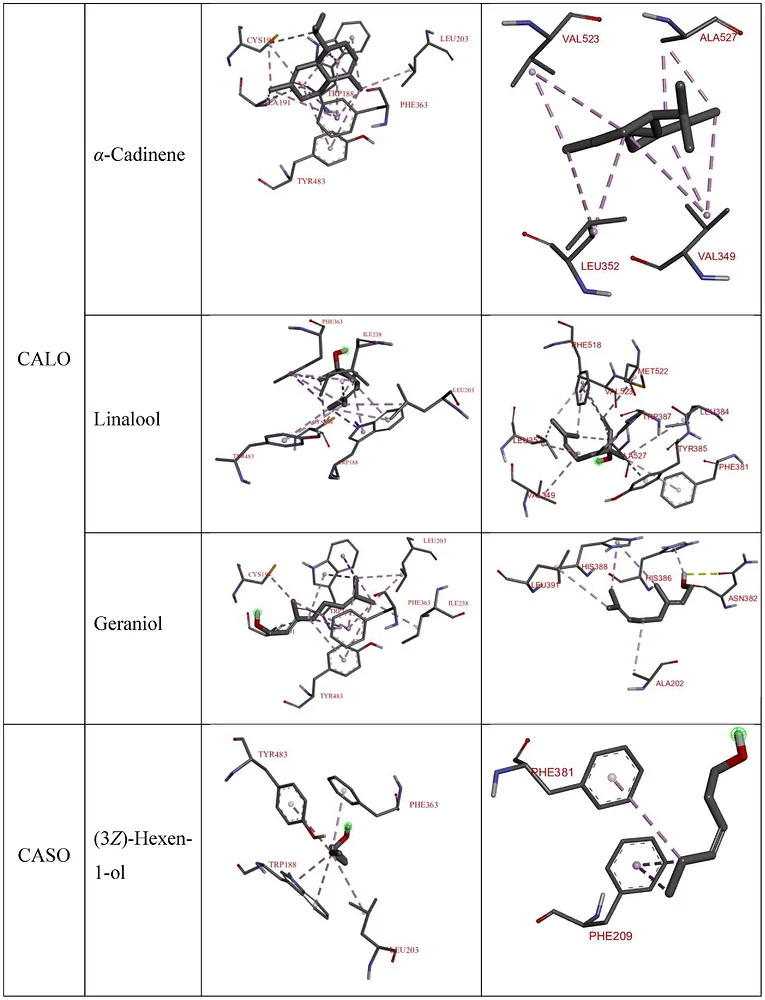

**Figure:**
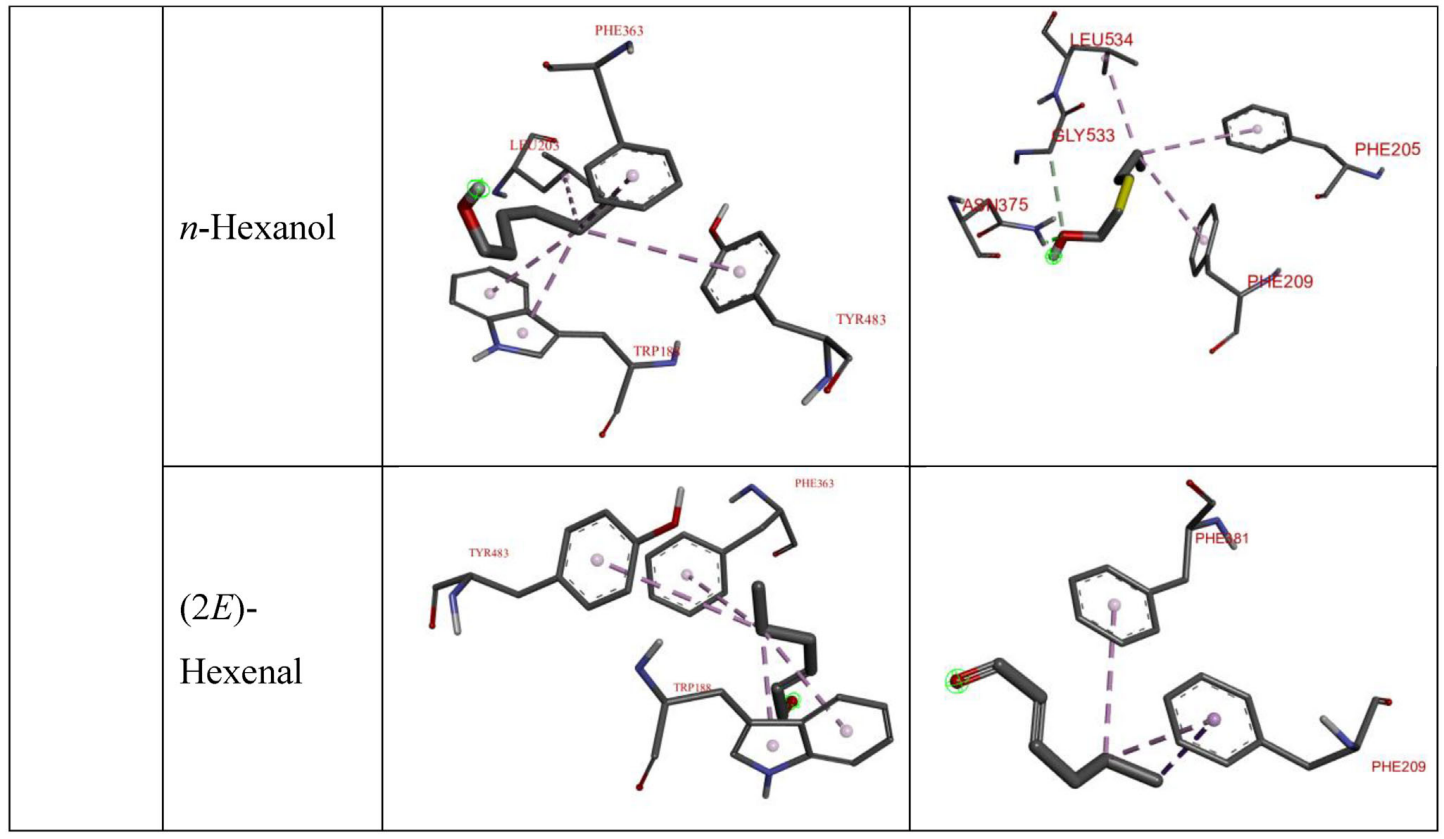

### 3.4 Anti‐Proliferation of Cancer Cells

The toxicity of EOs from *C. argentea* against several cancer cells, including Jurkat, A549, and Huh7, was evaluated using an MTT assay. The results demonstrated that Jurkat was more sensitive to CASO and CALO, while A549 was more sensitive to CASO (Figure 5). All EOs from *C. argentea* seemed less toxic to Huh7 cells. Among the tested EOs, CASO was the most effective in anti‐proliferation of cancer cells, especially with an IC_50_ on Jurkat cells of 7.39 ± 0.29 µg/mL. The positive control, ellipticine, was toxic to all cells, with IC_50_ values ranging from 0.32 ± 0.02 to 0.42 ± 0.03 µg/mL.

**FIGURE 5:**
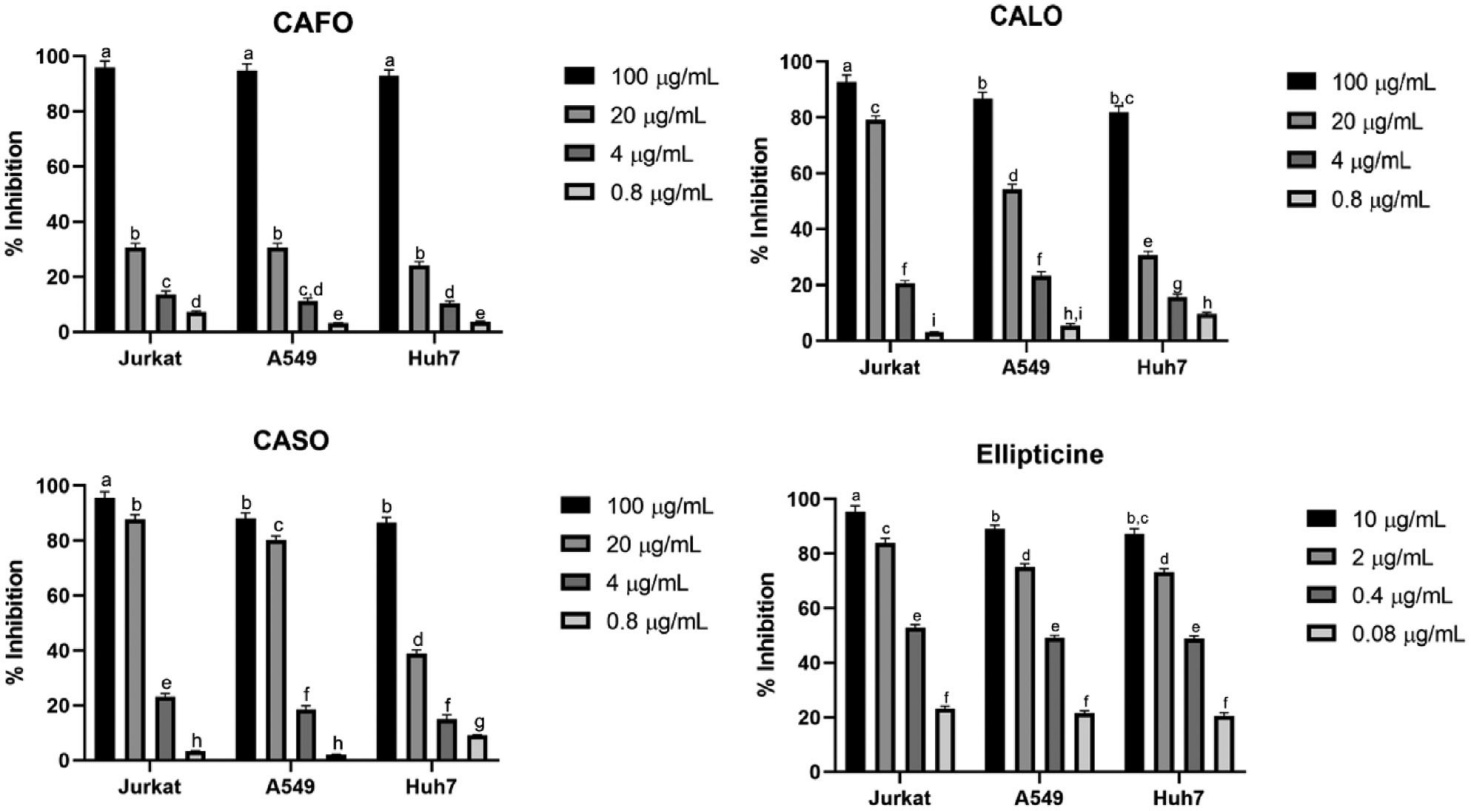
The cytotoxicity to cancer cells of *C. argentea* EOs. Ellipticine was used as a positive control. One‐way ANOVA with Tukey's multiple comparison was used; different letters represent significant differences.

A study of Joanna Kubik found that water and methanol–water extracts of *C. argentea* showed toxicity to 13 different cancer cells, with IC_50_ ranged from 64.53 to >125 µg/mL [42]. Compared to this result, the EOs in our study seemed more effective, with IC_50_ values ranging from 7.39 ± 0.29 to 33.07 ± 2.74 µg/mL in the tested leukemia, lung, and liver cancer cells. The water extract of *C. argentea* leaves was able to inhibit the proliferation of HepG2 cells with an IC_50_ of 112.24 µg/mL [43]. The major components of sesquiterpenes, monoterpenes, and diterpenes may be responsible for the cytotoxicity of EOs [44, 45]. Monoterpenes reduced the growth of cancer cells by inducing the apoptosis pathway by inhibiting anti‐apoptotic proteins such as Bcl‐2, Bcl‐xL, enhancing cytochrome C, Bax, and Bak; increasing ROS reaction, and disrupting the cell cycle via targeting cyclin D or CDK4 [46]. Many compounds in EOs of *C. argentea*, including δ‐cadinene, palmitic acid, geraniol, and linalool, have been demonstrated to be anti‐cancer agents [47, 48, 49, 50].

## 4 Conclusion

For the first time, EOs from stems, leaves, and flowers of *C. argentea* were isolated and analysed for chemical composition and bioactivity. The results indicated that the major constituents of CAFO were palmitic acid (16.93%), *cis*‐phytol (14.97%), and hexahydrofarnesyl acetone (13.71%), while those in CALO were *α*‐cadinene (44.42%), *cis*‐phytol (20.73%), and linalool (8.86%). (*3Z*)‐hexen‐1‐ol (41.90%), *n*‐hexanol (38.52%), and (*2E*)‐hexenal (7.31%) were the main compounds in CASO. CASO effectively inhibited nitric oxide production, exhibited cytotoxicity to cancer cells in vitro, and bound to inflammatory proteins in silico. These findings provide preliminary evidence of anti‐inflammatory and cytotoxic potential of *C. argentea* EOs and warrant further mechanistic and in vivo studies. In particular, the effects of EOs on the expression and release of pro‐inflammatory cytokines should be evaluated using techniques such as real‐time PCR and Western blotting. Furthermore, the underlying mechanisms responsible for the anti‐inflammatory effects should be elucidated. Additionally, further investigations are needed to elucidate their potential anticancer mechanisms, including their effects on apoptosis, cell proliferation, cell‐cycle regulation, mitotic checkpoints, and metastasis‐related pathways.

## Author Contributions

G.B.T., T.V.P., and B.H.D.: original draft preparation. G.B.T., T.V.P., T.X.H., N.H.N., and B.H.D.: methodology. B.H.D.: supervision, writing – review and editing.

## Conflicts of Interest

The authors declare no conflicts of interest.

## Supporting information




**Supporting File**: cbdv71885‐sup‐0001‐SuppMat.docx

## Data Availability

Data will be available on request due to privacy/ethical restrictions.
