# Supplementary material for: Comparison of Chemical Components, Anti‐Inflammatory Effect and Cytotoxicity to Cancer Cells of Essential Oils From Different Parts of Celosia argentea
Source: Chem Biodivers. 2026 Oct 30;23(11):e71885. doi: 10.1002/cbdv.71885 (PMC13721139; doi:10.1002/cbdv.71885)
Supplement: Supplementary file 1 [file CBDV-23-e71885-s001.docx]

**Comparison of chemical components, anti-inflammatory effect and cytotoxicity to cancer cells of essential oils from different parts of *Celosia argentea***

Short title: Bioactivities of *Celosia argentea* essential oil

Gia-Buu Tran^1^, Ty Viet Pham^2^, Thao Xuan Hoang^3^, Nguyen Hoai Nguyen^4^, Bich Hang Do^1,*^

**Supplementary**

Table S1. RMSD values of redocking

|  | **Co-ligand** | **RMSD** | **Verdict** |
| --- | --- | --- | --- |
| **iNOS** | H4B | 1.813 | Pass (<2 Å) |
| **COX-2** | ACD | 1.869 | Pass |

|  |  |
| --- | --- |
| a | b |

**Figure S1**. Overlay of the co-ligand and re-docked ligand at the binding sites of (a) iNOS and (b) COX-2. The original co-ligand is shown in blue, while the re-docked ligand is shown in pink.
